# The Feasibility of Haar Feature-Based Endoscopic Ultrasound Probe Tracking for Implanting Hydrogel Spacer in Radiation Therapy for Pancreatic Cancer

**DOI:** 10.3389/fonc.2021.759811

**Published:** 2021-11-04

**Authors:** Ziwei Feng, Hamed Hooshangnejad, Eun Ji Shin, Amol Narang, Muyinatu A. Lediju Bell, Kai Ding

**Affiliations:** ^1^ Department of Electrical and Computer Engineering, Johns Hopkins University, Baltimore, MD, United States; ^2^ Department of Radiation Oncology and Molecular Radiation Sciences, Johns Hopkins University School of Medicine, Baltimore, MD, United States; ^3^ Department of Biomedical Engineering, Johns Hopkins University School of Medicine, Baltimore, MD, United States; ^4^ Department of Gastroenterology, Johns Hopkins University School of Medicine, Baltimore, MD, United States

**Keywords:** endoscopic ultrasound (EUS), probe tracking, hydrogel spacer, pancreatic cancer, Haar feature, radiation therapy

## Abstract

**Purpose:**

We proposed a Haar feature-based method for tracking endoscopic ultrasound (EUS) probe in diagnostic computed tomography (CT) and Magnetic Resonance Imaging (MRI) scans for guiding hydrogel injection without external tracking hardware. This study aimed to assess the feasibility of implementing our method with phantom and patient images.

**Materials and Methods:**

Our methods included the pre-simulation section and Haar features extraction steps. Firstly, the simulated EUS set was generated based on anatomic information of interpolated CT/MRI images. Secondly, the efficient Haar features were extracted from simulated EUS images to create a Haar feature dictionary. The relative EUS probe position was estimated by searching the best matched Haar feature vector of the dictionary with Haar feature vector of target EUS images. The utilization of this method was validated using EUS phantom and patient CT/MRI images.

**Results:**

In the phantom experiment, we showed that our Haar feature-based EUS probe tracking method can find the best matched simulated EUS image from a simulated EUS dictionary which includes 123 simulated images. The errors of all four target points between the real EUS image and the best matched EUS images were within 1 mm. In the patient CT/MRI scans, the best matched simulated EUS image was selected by our method accurately, thereby confirming the probe location. However, when applying our method in MRI images, our method is not always robust due to the low image resolution.

**Conclusions:**

Our Haar feature-based method is capable to find the best matched simulated EUS image from the dictionary. We demonstrated the feasibility of our method for tracking EUS probe without external tracking hardware, thereby guiding the hydrogel injection between the head of the pancreas and duodenum.

## Introduction

Pancreatic cancer is the fourth most common cause of cancer death in both sexes in the United States. Perhaps more compelling, it is the most devastating cancer in the United States with the lowest 5-year relative survival rate of 9% (1). Furthermore, only a minority of cases representing resectable diseases have a chance for long-term survival. In contrast, one-third of cases do represent borderline resectable or locally advanced pancreatic cancer (BR/LAPC). Even if an aggressive therapy combining chemotherapy with radiation can be recommended for improving patients’ life quality in LAPC cases, the median survival is only extended to 9-15 months (2, 3). Previous autopsy studies proved that 30% of the patients died because of locally destructive diseases (4). Therefore, local control and delaying local progression are important for improving morbidity and extending the survival period for pancreatic cancer patients.

According to a previous study about dose escalation, the outcome with single fractions in 25 Gy or five fractions in 33 Gy were promising for leading a better local tumor control and delaying local progression. Furthermore, some researchers, recently, tested the dosimetric feasibility of implementing dose escalation with intensity-modulated radiation therapy (IMRT) with 67.5 Gy in 15 fractions prescription dose and stereotactic body radiation therapy (SBRT) with 50 Gy in 5 fractions prescription dose (5). Additionally, researchers demonstrated that the overall survival (OS) and local-regional recurrence-free survival (RFS) could be significantly improved after dose escalation during consolidative chemoradiation (6). However, even with the wide implementation of proton therapy and better optimization method (7), the challenge and barriers to implementing these dose escalation strategies involve the proximity and inherent radiosensitivity of the gastrointestinal tract, particularly the duodenum, which is directly adjacent to the head of pancreas (HOP). Plus, the motion of abdominal organs caused by breathing increased the risk of these radiosensitive organs in radiotherapy (8, 9).

In that hydrogel is capable of sparing organs at risk (OARs) from radiation targets, hydrogel injection is a potential solution for reducing the radiation dose received by radiosensitive OARs, thereby sparing them during dose escalation treatments. The utility and outcome of this technique have been evaluated in the treatment of prostate, head and neck, and gynecologic cancers (10–14). By increasing the space between the rectum and the prostate, the radiation dose received by the rectum was reduced, thereby improving the safety of radiation treatment and quality of life (10). Similarly, a previous study in gynecologic malignancy patients proved that hydrogel injection resulted in a significant reduction in the dose delivered to the rectum (13). Furthermore, our previous study has assessed the feasibility of injecting a similar injectable absorbable radiopaque hydrogel spacer (TraceIT, Augmenix, Bedford, MA) between the HOP and the duodenum *via* endoscopic ultrasound (EUS) guidance in human cadaveric specimen experiments (15–18). This TracelT is made up of a hydrogel paste that generates a bleb of particles at the needle tip upon injection. As previous research and the development report showed, the bleb maintains its 3-dimensional structure for three months and is absorbed after seven. We demonstrated the stability, safety, and efficacy of using this hydrogel in pancreatic cancer by creating sufficient space to protect the duodenum and to enhance the potential for dose escalation (16, 19). At present, our group is proceeding with a clinical trial to access the utility of placing a hydrogel spacer between the HOP and the duodenum in BR/LAPC pancreatic cancer cases by EUS guidance without invasion of the duodenum (15).

**Figure 1:**
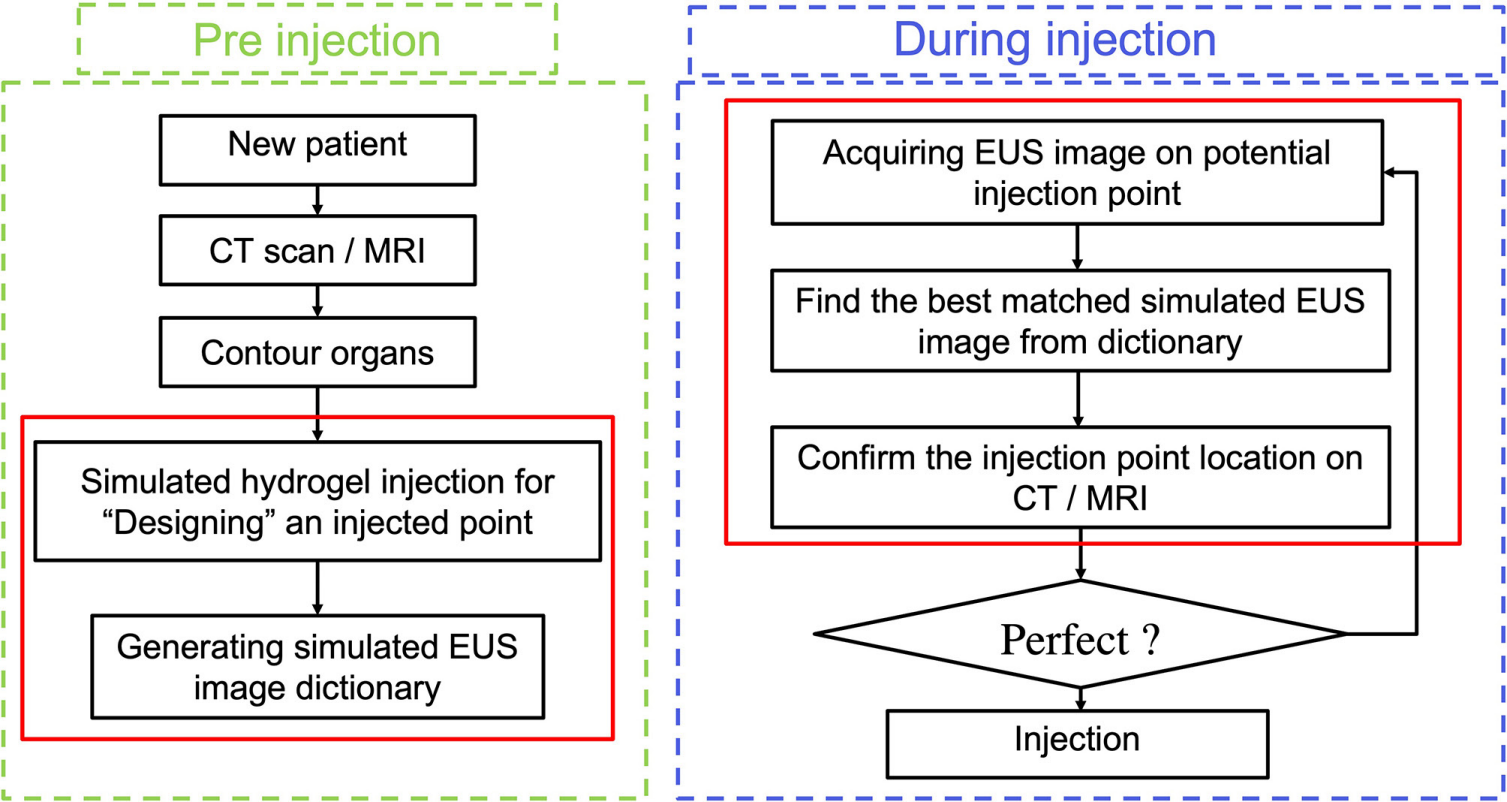
Ideal workflow of injecting hydrogel.

However, the efficacy of utilizing hydrogel and the accuracy of injecting it can be compromised due to the uncertainty of how much hydrogel is needed and where the optimal hydrogel injected points should be along with the HOP-duodenum interface. For normalizing and perfecting the EUS injection procedure, we proposed an ideal injection workflow in **Figure 1**, including a prediction of separation for anticipating how much hydrogel is injected (20), injection planning, and execution of injection for guiding hydrogel injection in an optimal injected point. As we proposed, before the injection process, injection planning was designed with an optimal injection point based on the anatomical relationship from diagnostic computed tomography or Magnetic Resonance (MR) images. The challenge of executing injection planning centers on how to track the endoscopic probe relative position to the CT (21) or MR images and where to place the probe in the designed injection point. In other words, the challenge is how to align the real-time EUS image with the diagnosed CT or MR images, thereby guiding the injection process to be executed as planned. The present study is mainly aimed to test the feasibility of our method in steps with red frames.

The existing technical solution for ultrasound guidance systems mainly relies on external tracking hardware, such as optical camera, electromagnetic tracking, or mechanical tracking hardware (22–25). These hardware techniques were used to compute consistency between real-time 2D/3D ultrasound images and diagnostic 3D CT or MR images. But owing to the calibration in the clinical procedure, the error caused by internal organs motion was ignored in this tracking method. Therefore, researchers proposed a variety of multi-modalities image registration methods to compensate that tracking errors. For example, Hu et al. (26) developed an automatic non-rigid feature-based registration between magnetic resonance and 3D transrectal ultrasound (TRUS) images (26). They extracted surface normal vectors of 3D US images by using a second-order Gaussian filtering approach, thereby reducing the system sensitivity to noise. In addition, for enhancing the accuracy of EUS navigation in the upper gastrointestinal (GI) system, Bonmati et al. (27) proposed a registration approach to simulate the initialization between pre-obtained CT images to EUS registration by registering landmarks to corresponding segmented anatomical structures (27). By testing different cost-functions (cross correction, mutual information, and gradient methods), Shi et al. (28) optimized the image registration for projecting mucosal disease contours to planning CT datasets by accomplishing rigid registration of optical endoscopy image and CT scans (28). The image registration between EUS and CT images remains a challenging task with low robustness and accuracy. This is owing to: a) the loss of image information of 3D CT data in 2D EUS images; b) the lack of paired anatomical landmarks on EUS/CT; c) the difference of grey level in some structures in CT and EUS images.

One potential candidate to address the challenges is using the Haar feature. Haar features are efficient to represent image information with fast performance. The previous implementations of the Haar feature focused on fingerprint compression, face reorganization (29, 30), and JPEG Image compression (31–33), all of which have promising accuracy for detecting objects. According to previous research, Haar features have a good capacity in distinguishing functions in cascades (29). Silva et al. (31) developed a dictionary-based 3D MR -2D EUS images initialization algorithm by extracting image Haar features to estimate the initialized pose. After initialization, they proposed a fast image-based 3D-2D registration by Powell’s method (31, 34). The results proved that these Haar features were an efficient representation for initialized pose utilization for guiding spinal intervention. Thus, these characteristics of Haar features makes it possible to overcome our previous challenges.

In our present study, we develop a Haar feature-based method for tracking EUS probe location on diagnostic CT or MRI without external tracking hardware to facilitate injecting hydrogel in designed injection points. Our methods included the pre-simulation section and Haar features extraction sections. **Figure 1** shows the overview of our proposed method. In the first step, the simulated EUS image set was generated based on anatomic information from CT or MRI images. Secondly, the efficient Haar features are extracted from simulated EUS images set to create a Haar feature dictionary. The probe relative position is estimated by searching the best matched Haar feature vector of the dictionary with Haar feature vectors of real EUS images. The utilization of this method was evaluated in endoscopic phantom, patient CT scan, and patient MR scan.

## Materials and Methods

### Methodology

#### Field II Simulation and Simulated EUS Dictionary

Field II is a free and open-access Matlab program for simulating ultrasound images by calculating the ultrasound field for both the pulsed and continuous wave case. This method is based on linear systems theory (35–37). When the transducer emits the signal as a Dirac delta function, the corresponding emitted ultrasound field is represented as a time function of a specific point in space according to the spatial impulse response. Thus, this field of different excitations is calculated by convolving the different excitation functions with the spatial impulse response. The detailed explanation and reasoning of this simulation method were published in previous publications (38–40).

**Figure 2:**
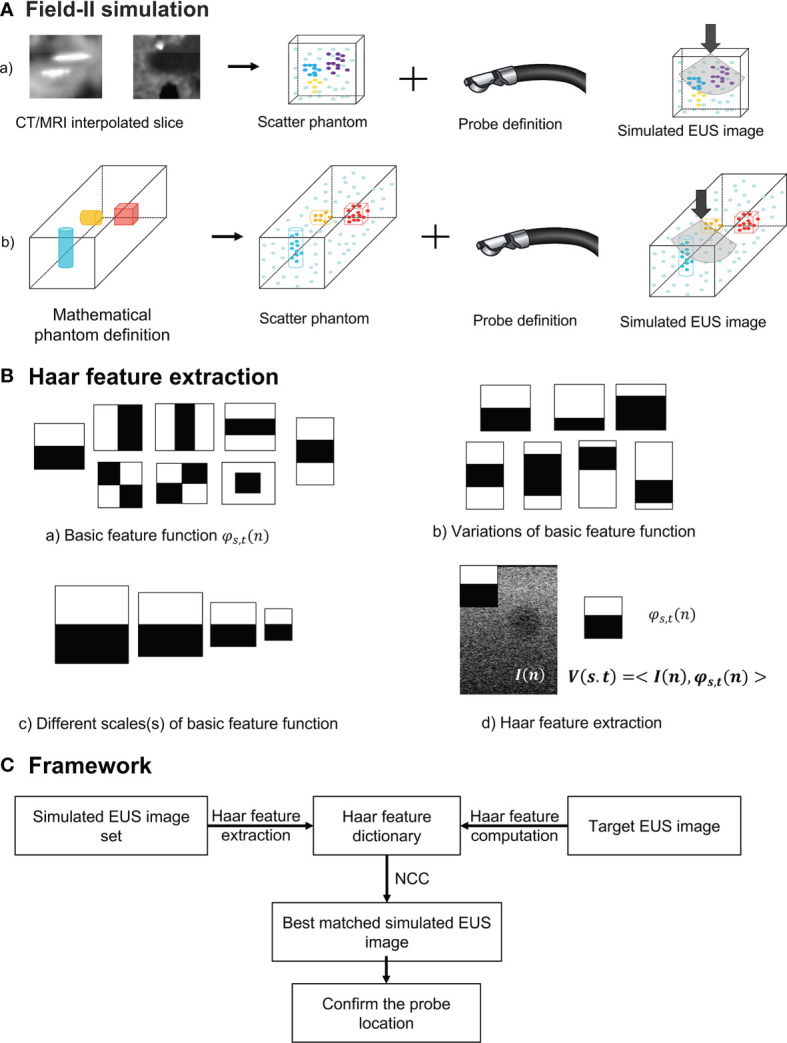
**(A)** The diagram of Field II simulation. **(B) (a)** Basic Haar feature function; **(b)** Two examples of variation of basic feature function; **(c)** Example of different scales of basic feature function; and **(d)** Computation of Haar feature extraction. **(C)** The overview of our proposed framework. NCC, normalized cross-correlation.

In our study, we used two different methods to generate a simulated scatter phantom. As **Figure 2A** row one shows, the first method is used for patient scans. We simulated anatomic scattered phantoms based on the interpolated CT/MR slice of the region of interest. The detail of this method is explained on Field II’s official website (http://fieldii.dk//?examples/kidney_example/kidney_example.html). The second method is applied for the phantom experiment. As **Figure 2A (b)** shows, we built a scatter phantom based on our endoscopic training phantom by defining geometric targets directly and assigning proper amplitudes for corresponding scatters. Then, the corresponding simulated EUS images were generated from the top center of the scatter phantom.

#### Haar Feature Extraction

To accurately and efficiently represent images features, we define eight basic Haar functions shown in **Figure 2B (a)** with scaling (s) and translation (t) parameters as:


(1) $$\varphi_{s,t}(n)=\varphi (2^{s}n-t)$$

Where *n* = (*n_x_*, *n_y_*) and *t* = (*t_x_*, *t_y_*) are spatial vectors representing x and y components. The translation parameter t defines the spatial location of the basic Haar feature function. According to previous research, these basic Haar functions are capable of capturing enough intensity patterns of images used for face detection and segmentation (31). Furthermore, the function with the relatively large scaling s is more sensitive to the detection of fine anatomical variation patterns with a large amount of computations. In contrast, by increasing this scaling parameter *s*, the corresponding basic function is able to detect large anatomical variations with fewer coefficients. We varied the scaling parameters in one interval for every basic Haar feature function [**Figure 2B (c)**]. On the other hand, as displayed in **Figure 2B (b)**, the other type of variation for every basic feature function is changing black and white block proportions. To improve the efficiency of computation, the integral image method proposed by Viola and Jones (29) is also implemented as the dot product of the basic Haar function and the image [**Figure 2B (d)**]:


(2) $$V(s,t)=\;<I(n),\varphi_{s,t}(n)>$$

where I(n) is the image intensity, and V is the Haar feature vector. Therefore, the Haar feature vector includes the response of different scales, translations, and variations of basic feature functions, which is enough for encoding images features. The Haar feature dictionary is formed by computing this Haar feature vector of each simulated 2D EUS image.

#### Overview of the Framework

The overview of our Haar features-based EUS imaging guidance is shown in **Figure 2C**. We tested the feasibility of this method on both the phantom and patient images. Firstly, we generated the simulated EUS image set of phantom or patient which consists of simulated EUS images from Field II. Then, Haar feature vectors of all simulated images were computed and extracted to form a Haar feature dictionary. Similarly, the Haar features vector of the target EUS image was computed as well.

As **Figure 2C** reveals, by calculating normalized cross-correlation (NCC) between the Haar feature vector of target EUS images with every vector within the dictionary, the best matched simulated EUS image was confirmed with the maximum NCC value. The NCC was calculated as follows:


(3) $$I_{match}=\underset{\left\{I_{sEUS_{i}}\right\}}{\arg\max}\frac{1}{N_{s}}\Sigma_{s=1}^{N_{s}}NCC(V_{hi}(s),h_{target}(s))$$

where *V_hi_* (*s*) is the *i*-th Haar feature vector from the dictionary, and *h_target_* (*s*) is the Haar feature vector of the target EUS image. *N_s_* is the number of feature coefficients at every scaling level (31), and *I_match_* is the corresponding best matched simulated EUS image. In different experiment, all simulated EUS images are paired with different kinds of 3D image modality, such as CT or MRI. Because we know the location of each simulated EUS image on paired 3D CT or MRI, the probe location is tracked and confirmed in the 3D image data.

### Experiments

#### Phantom Experiments

##### Simulated EUS Image and Interpolation CT Scans

**Figure 3:**
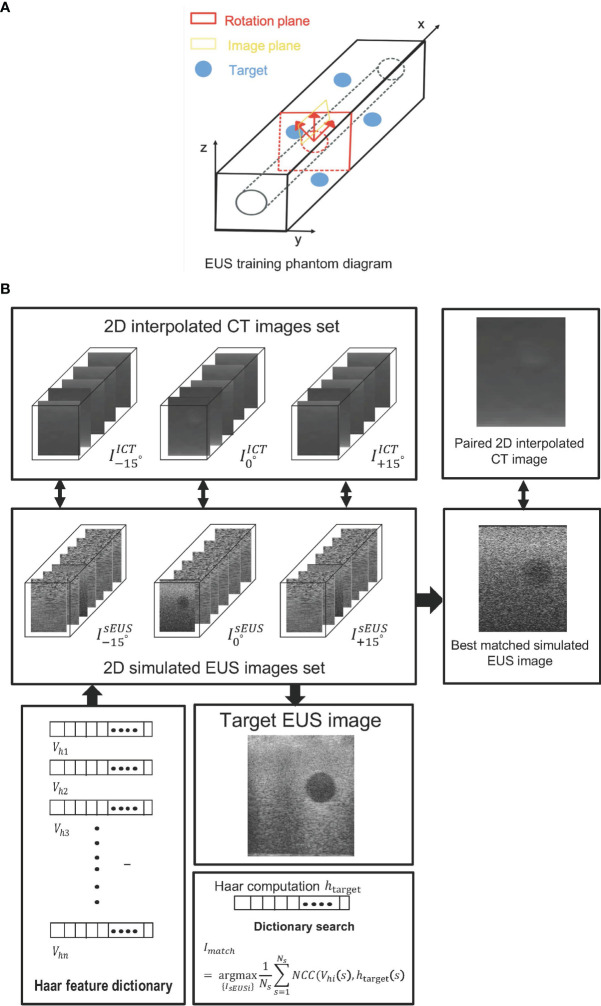
**(A)** The EUS training phantom and CT interpolated plane diagram. **(B)** The workflow of a phantom experiment.

**Figure 3** depicts the interpolated computed tomography (ICT) and corresponding simulated EUS images of our EUS training phantom (ATS Laboratories, Model GIETP). This phantom includes four echogenic sphere targets with a 5-mm radius, which are randomly distributed in a soft rubber-based tissue-mimicking material. A scan channel with a 25-mm radius is in the center of this phantom. This phantom was scanned with a Philips Big Bore 16-slice CT simulator (120 kVp, 1000 mAs/slice, collimation 16 x 1.5-mm, pitch 0.059, rotation time 0.44 s, FOV 600 mm, ultrafast recon kernel, 3-mm slice thickness, 3-mm increment, and standard filter). CT scan datasets were interpolated into a 1-mm slice thickness based on Matlab (Mathwork, Inc, R2020.a). All ICT scans were generated based on these interpolated datasets. Four targets were manually contoured from the CT scan based on Velocity software (Varian Medical Systems, Inc).

One set of real-time EUS images was obtained with a linear endoscopic probe with 128 elements and reconstructed based on the Vantage 128 system (Verasonics Inc., WA, USA). This real-time EUS image was used as the target EUS image, and we generated one corresponding simulated EUS image and interpolated CT scan as ground truth to test our method.

Based on the coordinate data of the targets’ contour, an artificial scatter phantom with four identical sphere targets was simulated using Field II. At every target center, as shown in **Figure 3A**, the $I_{0^{\circ }}^{sEUS}$ represented simulated EUS image set simulated by shifting the image plane along the center of each target axis at 5-mm intervals. Similarly, the $I_{\pm 15^{\circ }}^{sEUS}$ represented simulated EUS image sets simulated by rotating image direction to ± 15, respectively. Thus, a 2D simulated US image set $\left(I_{0^{\circ }}^{sEUS}\text{ and }I_{\pm 15^{\circ }}^{sEUS}\right)$ including 30 images was created for every target. The corresponding 2D interpolated CT image $\left(I_{0^{\circ }}^{ICT}\text{ and }I_{\pm 15^{\circ }}^{ICT}\right)$ sets were created in the same rotated degree and image plane. In the Field II simulation process, we defined the parameters of the endoscopic probe exactly as the endoscopic probe, including a center frequency of 7.5 x 10^6^ Hz and a width and height of every element at 0.29 mm and 0.41 mm, respectively. Before computing the Haar feature dictionary, all simulated EUS images are smoothed by a 5 x 5 median filter for removing scattered noise.

**Figure 3B** shows the workflow of phantom experiments. Each simulated EUS image was registered and paired with a corresponding interpolated CT image in the same location. The simulation of the EUS images process and the interpolation process were performed on the Field II package and our lab software, respectively. The Haar feature vectors of every simulated EUS image consisted of the Haar feature dictionary. By calculating the NCC value between the Haar feature vector of the target EUS image and each vector in the dictionary, the best matched simulated EUS image was found and picked from the simulated EUS set, thereby finding the corresponding paired ICT slice and tracking probe location.

In addition, we tested the matching accuracy and efficiency with a different number of basic Haar feature functions and scale parameters based on phantom experiment data. In this test, the experimental procedure was the same as the previous one but with a different number of basic Haar feature functions or changing the scale parameters.

#### Patients Experiments

##### Patient’s Experiment Based on CT Scans

For assessing the feasibility of our method on more complicated image data from a real patient, we used our previous patients who were injected with hydrogel spacer at our institution before radiation therapy. Six EUS record videos during the injection process were collected and real EUS image data were generated by extracting frames from these videos. After injection of the hydrogel, CT simulation of this patient (Philips Brilliance Big Bore CT; 3-mm slice thickness, 120 kVp, 200 mA, a 60-cm field of view) was performed. 10 potential injected points located in different slices were selected by the clinician, and corresponding ICT slices of each potential injected point were created. Every ICT slice has different directions which are different from the traditional CT view (axial, sagittal, and coronal) for mimicking the various views of EUS images. The tumor region was represented as hypoechoic mass whereas no clear edge of the tumor was observed based on CT scans. Thus, according to the tumor contour, we applied a pre-processing step of ICT scans for converting tumor pixels’ grey level into black and generated these pre-processing ICT.

**Figure 4:**
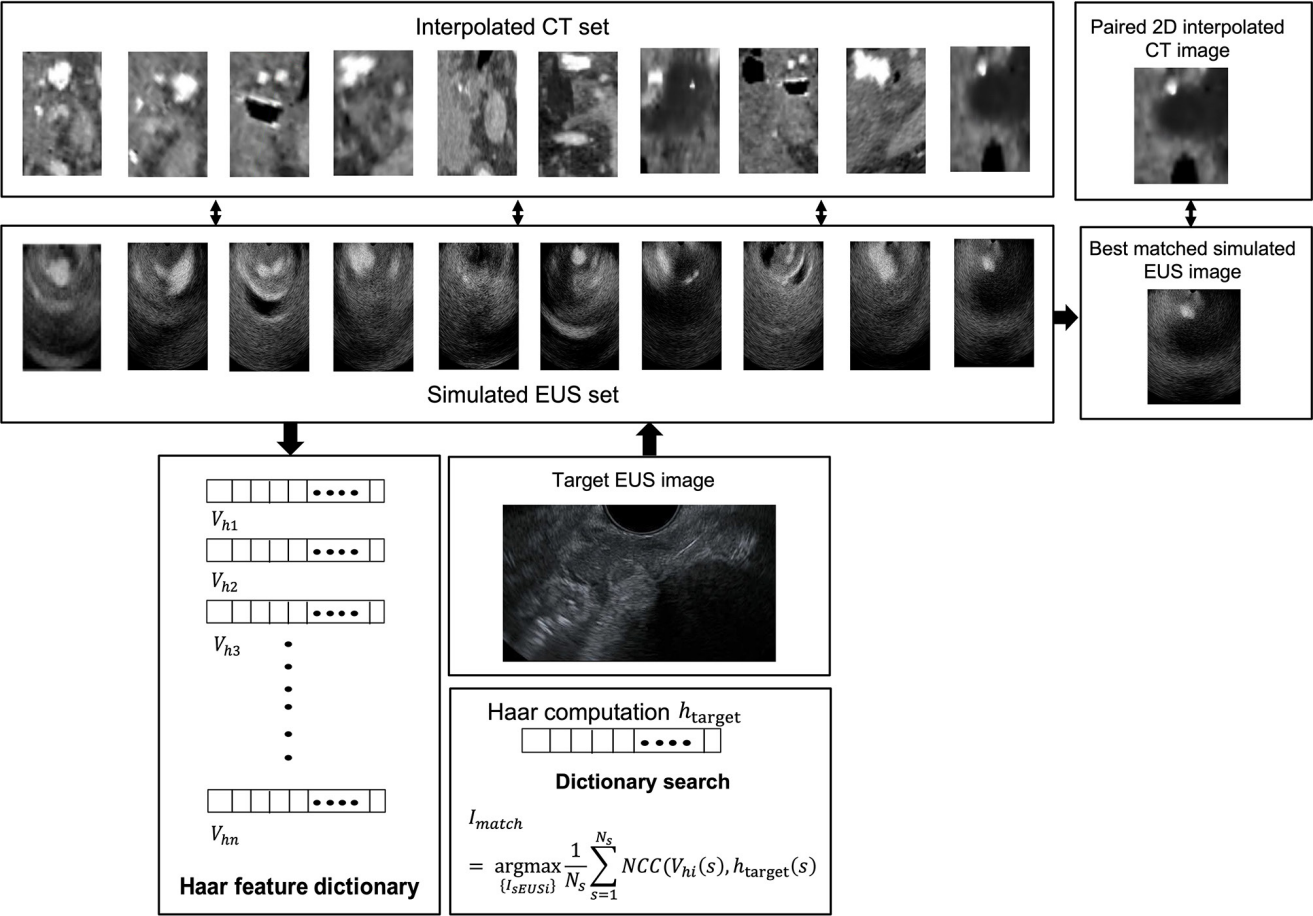
The workflow of patient’s CT experiment.

A simulated EUS data set was generated by simulating ten EUS images based on pre-processing ICT slices. Specifically speaking, the anatomic phantoms were created by drawing a bitmap image of the scattering strength of the region of interest. In this case, this bitmap determines the factor multiplied with the scattering amplitude generated from the Gaussian distribution, thereby modeling the difference in the density and speed of sound perturbations in the tissue. A curvilinear array with 159 elements in 91.1 mm radius was defined and the simulated EUS images consisted of 128 scanlines. **Figure 4** showed the workflow of the patient’s CT experiment. One real EUS image was selected by the clinician as the target EUS image. By extracting Haar feature vectors from simulated EUS images and target EUS images, and computing the NCC value between every simulated EUS image with target one, a best matched simulated EUS image was obtained with a known location on the ICT scan. Therefore, the probe position of the real target EUS image can be confirmed as the location of the best matched simulated EUS image.

##### Patient Experiment Based on MR Images

**Figure 5:**
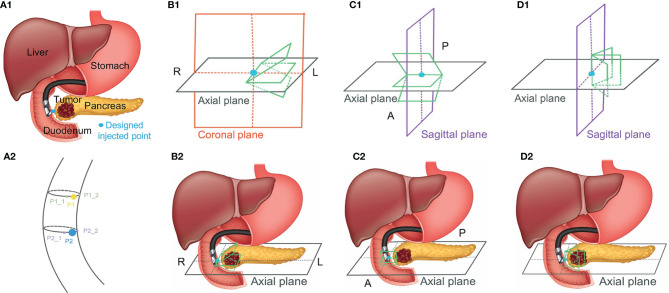
The simulated injected point and interpolation of MRI image. R, L, P, and A represent right, left, posterior, and anterior, respectively.

**Figure 6:**
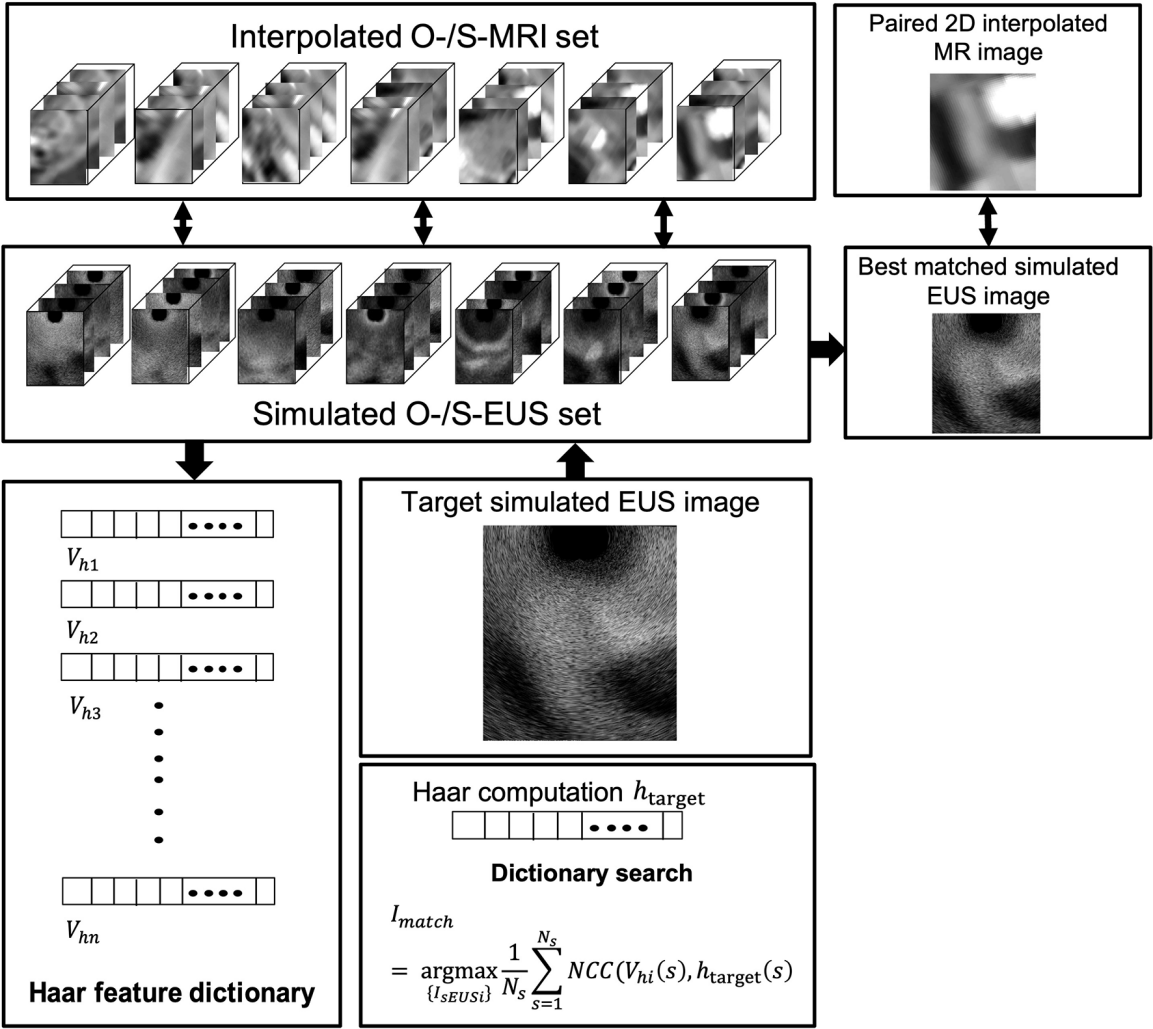
The workflow of patient’s MRI experiment.

Because MR images have a better contrast around the HOP, we further tested the feasibility of our method based on MR image data. We defined the MR images of our previous patients acquired before hydrogel injection at our institution as original pre-MR images (O-MRI). Patient MR images were performed on a 1.5T clinical MR scanner (Signa Artist, GE Healthcare, Wauwatosa, WI, US). We created two simulated injected points based on different MR image slices. Then, we simulated hydrogel injection at these two simulated injected points, named P1 and P2, and generate simulated post-MR images (S-MRI). As **Figure 5** A2 shows, the yellow and blue points are two simulated injected points in different axial slices. P1_1 and P1_2, P2_1 and P2_2 are the extra two nearby points around simulated injected points, respectively. All the simulated injected points were selected based on axial CT scan which duodenum and HOP were adjacent to each other. The detail of this simulation of injection was published in the previous paper from our lab (41). We generated 7 interpolated MRI slices on every simulated injected point. As **Figure 5**, B1 and C1 show, for each simulated injected point, we interpolated four MR image slices by rotating image planes ±15° around the axial plane in the patient right-to-left direction (**Figure 5**, B1, R to L) and the posterior-to-anterior (**Figure 5**, C1, P to A) direction, respectively. These simulated EUS images were aimed to mimic the EUS images obtained like radial EUS scanning. Additionally, as **Figure 5**, D1 shows, the other three interpolated MRI slices were perpendicular to the axial plane. The middle-interpolated slice was defined as a simulated injected image plane from point to the HOP and the rest of the two interpolated slices were generated by shifting ±15° around it. These simulated EUS images were used to mimic the EUS images collected as in linear EUS scanning. Therefore, two interpolated MRI sets were created based on OP-MRI and SP-MRI data sets and each of them included 42 interpolated MR images. As **Figure 6** shows, the corresponding simulated EUS data sets were generated by simulating EUS images based on interpolated O-MRI and S-MRI data sets. The simulation process was the same as patient CT experiments, including probe definition and generation of scattering phantom.

We did two validation experiments. In the first experiment, we picked one simulated EUS image (from P2 simulated injected point with **Figure 5**, B1 –15° interpolated angle) from O-MRI simulated EUS image set as the target EUS image. We assume this target EUS image is obtained before the injection process and try to track the probe location of this target EUS image. So, we searched the best matched simulated EUS image of this target EUS image from the O-MRI simulated EUS image set. In the second experiment, we picked one simulated EUS image at the same simulated injected position in the previous target EUS image (from P2 simulated injected point with **Figure 5**, B1 –15° interpolated angle) from S-MRI simulated EUS data set as the target EUS image. In this case, our scenario is to simulate this target EUS image obtained during the injection process and try to track the probe location in real-time for the guidance injection process. Thus, we searched the best matched simulated EUS image of this target EUS image from O-MRI simulated EUS image data sets.

## Results

**Figure 7:**
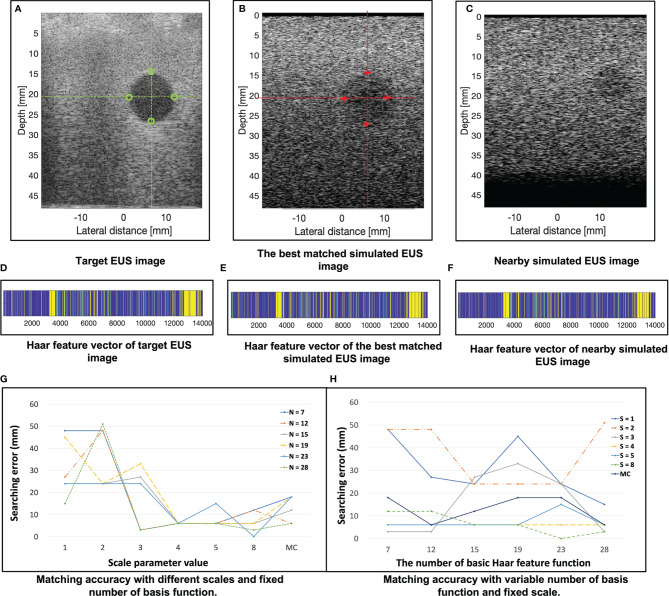
**(A–C)** show the target EUS image, the corresponding best matched simulated EUS image, and one simulated EUS image with a different target nearby the best matched one. Four marker points are measured in the target EUS image and the best simulated US image. The green circles and red crosses represent the marker of the target EUS image and best matched simulated EUS image, respectively. **(D–F)** show the Haar feature vector of target EUS image, best matched simulated EUS image, and nearby simulated EUS image, respectively. **(G)** plots the results of matching accuracy with different scales and fixed number of basis functions. N is the number of basis functions. MC is a combination of multiple scales. **(H)** plots the results of matching accuracy with a variable number of basis functions and fixed scales. S is the scale of the basis function. MC is a combination of multiple scales. The error is calculated as the distance error of the four marker points of each target between the target EUS image and the corresponding best matched EUS image.

In the 121 simulated EUS images with 18,801,134 scatters, the best matched simulated EUS image is the 19^th^ in the $I_{0^{\circ }}^{SUS}$ . The results are shown in **Figure 7**. **Figures 7A–C** show the target EUS image, the corresponding best matched simulated EUS image, and one simulated EUS image with a different target nearby the best matched one. Four marker points are measured in the best simulated EUS image and the target EUS image. The green circles and red crosses represent the markers of best matched simulated US image and target EUS image, respectively. The locations of four marker points on the target are obtained. Because we did not have the ground truth location of the target EUS image, the distance errors between these four marker points are used to assess the process for searching best-matched result. In the x-axis, the errors of these four markers between the target EUS image and the best matched EUS simulated image are -0.36 mm, -0.71 mm, -0.71 mm, and 0.07 mm, respectively. In the y-axis, the errors of these four markers between the target EUS image and the best matched US simulated image are 0.468 mm, 0.80 mm, 0.91 mm, and 0.91 mm, respectively. **Figures 7D–F** show the Haar feature vector (1*14027) of the target EUS image, best matched simulated EUS image, and nearby simulated EUS image, respectively.

**Figure 7G** plots the results of matching accuracy with different scales value and a fixed number of basis functions. N is the number of basis Haar feature functions. MC is a combination of multiple scales values. **Figure 7H** plots the results of matching accuracy with a variable number of basis functions and fixed scales. S is the scale value of the basis Haar feature function. MC is a combination of multiple scales. The locations of four marker points on the sphere target are obtained. The error was measured as the four marker points’ distance errors of each target between the target EUS image and the corresponding best matched EUS image. As **Figures 7G, H** show, with a fixed basic Haar feature function, the matching accuracy will be improved by increasing scales. That is because the lower image quality of simulated EUS images did not have smooth circle edges. It is better to use large scales of basis function to detect the large gray level variation. According to **Figure 7H**, there is no significant relationship between matching error with the different number of basic Haar feature functions (fixed scales).

**Figure 8:**
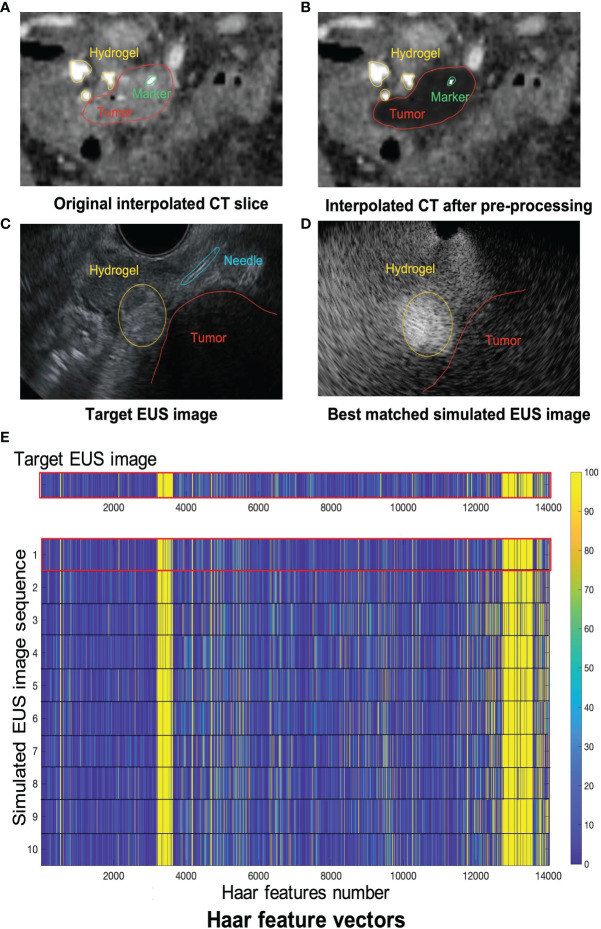
**(A, B)** show one example of ICT and pre-processing ICT, respectively. Red, yellow and green contours represented tumor, hydrogel, and tumor marker, respectively. The target EUS image and the best matched simulated EUS image are shown in **(C, D)**, respectively. Red, yellow, and blue contours represented tumor, hydrogel, and injected needle, respectively. **(E)** shows the Haar feature vector comparison between the target EUS image and all 10 simulated EUS images. The Haar feature vector in the red frame is calculated based on the best matched simulated EUS image.

**Figure 8** shows one example of ICT and corresponding pre-processing ICT. In the ICT, the tumor, as red contour showed, has a similar grey level with around tissue (**Figure 8A**). **Figure 8B** shows the result of the tumor region after pre-processing which was converted as a “hypoechoic” organ (red contour). A white dot (green contour) in the **Figures 8A** and 8B within the tumor is a marker for eliminating position error during radiotherapy. There are three hydrogel clusters in the ICT and PCT with yellow contours (**Figures 8A, B**). The corresponding simulation phantoms are created for EUS simulation based on these pre-processing ICT slices. **Figure 8C** shows one frame of real EUS image selected by a clinician as target EUS images to test our method. A tumor (red contour), one hydrogel cluster (yellow contour), and injected needle (blue contour) can be seen in this target EUS image. The tumor is presented as a hypoechoic region at the bottom right of the image. By applying our proposed method, the best matched simulated EUS image is found according to the maximum NCC value. **Figure 8D** shows the corresponding best matched simulated EUS image in our simulation datasets. The tumor and hydrogel clusters are contoured in red and yellow, respectively. **Figure 8E** shows the Haar feature vectors (1*14027) of the target EUS image and all 10 simulated EUS images. Different colors represented different values. The x-axis represented the feature number. The Haar feature vectors of the real EUS image and the corresponding best-matched image were shown in the red frame (**Figure 8E**).

**Figure 9:**
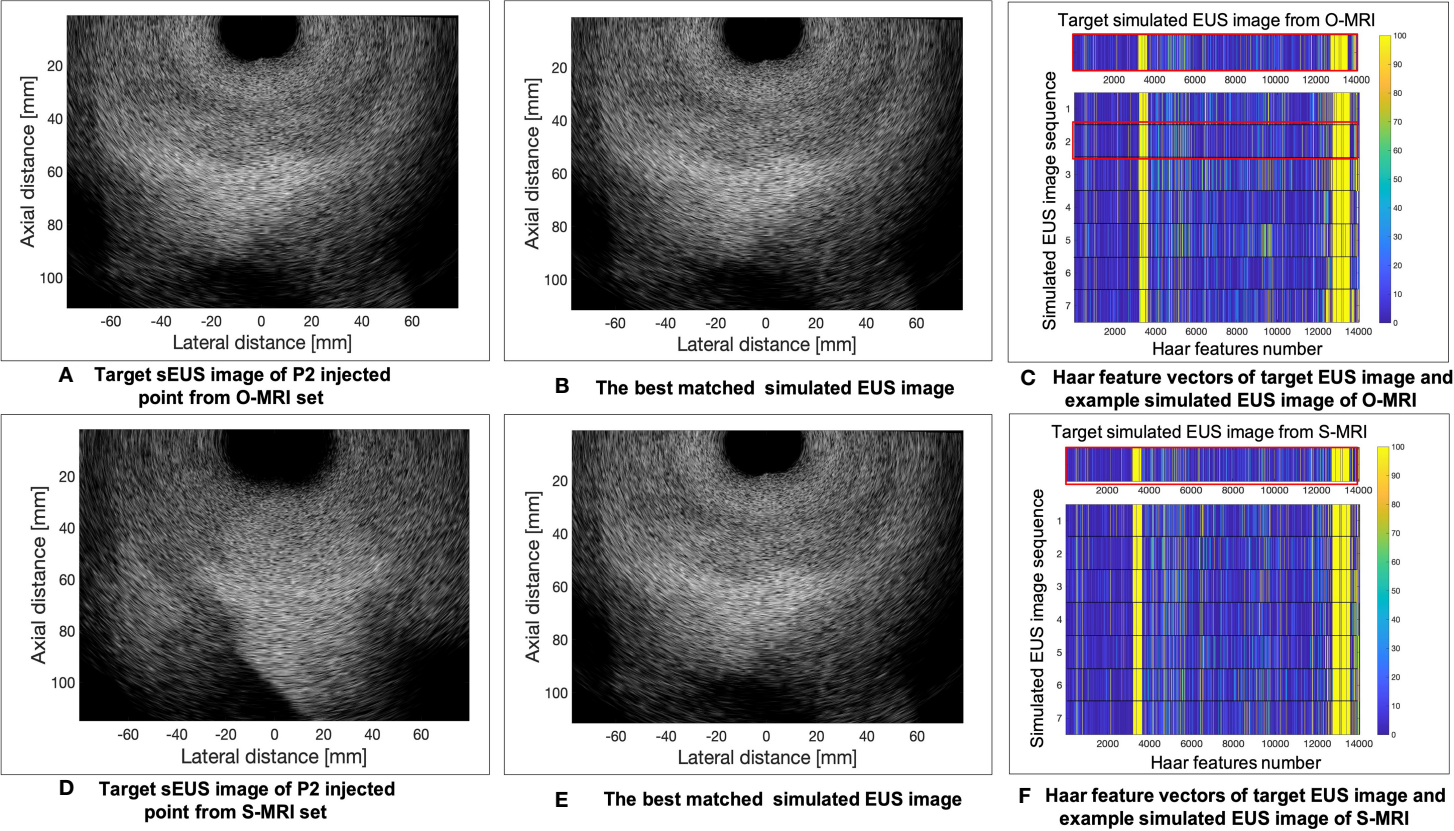
The example results of the MRI patient experiment. **(A)** shows the target simulated EUS image which was chosen from O-MRI simulated EUS image set at the P2 simulated injected point with . B1 –15° interpolated direction. **(D)** shows the target simulated US image from S-MRI at the same position. **(B, E)** show the corresponding best matched simulated EUS images found in the O-MRI simulated EUS set. **(C, F)** show the Haar feature vectors comparison between target EUS images and simulated EUS images from the same potential injected points. The Haar feature vectors of the target EUS image and corresponding best matched simulated EUS image was in the red frame.

The results of the MRI patient experiment are shown in **Figure 9**. **Figure 9A** shows the target simulated EUS image which is chosen from O-MRI simulated EUS image set at the P2 simulated injected point with **Figure 5**, B1 -15° interpolated direction. **Figure 9D** shows the target simulated EUS image from S-MRI at the same position. **Figures 9B, E** show the corresponding best matched simulated EUS images found in the O-MRI simulated EUS image set. Our method is capable to find the best matched simulated EUS image both before injection and during the injection process, thereby confirming the probe location. But if the target EUS image is chosen as the simulated EUS images in the same simulated injected point with different interpolated directions, the best matched simulated EUS images were found with error interpolated direction.

**Figures 9C, F** show the Haar feature vectors comparison between target EUS images and simulated EUS images from the same potential injected points. The target EUS image shows in **Figure 9C** is chosen from O-MRI simulated EUS image set at the P2 simulated injected point with **Figure 5**, B1 -15° interpolated direction. The Haar feature vectors of simulated EUS images are based on O-MRI from the same simulated injected point. The target EUS image shows in **Figure 9F** is chosen from S-MRI simulated EUS image set at the P2 simulated injected point with **Figure 5**, B1 -15° interpolated direction. The Haar feature vectors of simulated EUS images are based on S-MRI from the same simulated injected point. The Haar feature vectors of best matched simulated EUS image is the second vector in **Figure 9C** for both these two targets simulated EUS images (red frame in **Figure 9C, F**).

## Discussion

We proposed a Haar feature-based method for tracking probe position on diagnostic CT/MRI scans in the hydrogel injection process between the HOP and duodenum. We tested our method on a phantom study and two patients’ experiments. Such a method can potentially increase the efficiency of hydrogel placement in common practice and obviate the need for external hardware for tracking EUS probe positions. The significance of our method is building a connection between 2D real-time EUS images and 3D pre-diagnosed CT/MRI images.

In previous research, we considered two possible risks of hydrogel injection (16). The first potential risk is about the side effects of muscularis propria after injection. Due to the unique anatomy between the HOP and duodenum, the hydrogel spacer injection process caused the injection within the muscularis propria of the duodenum in our cadaveric specimens. The second possible risk is disrupting and disseminating microscopic disease between the HOP and duodenum. Thus, we planned to evaluate and test whether the hydrogel injection causes the microscopic disease extent based on the histopathologic analysis of the resected interface between the HOP and duodenum. In this case, for better understanding the possible side effects of injection before expanding spacer application to clinical trials, it’s important to control hydrogel injected in a specific location, mark and record this specific location in three-dimensional image data, and then investigate and identify whether this location is safe to place spacer with low risks. This is another potential application of our proposed method.

Besides these risks, there are two main uncertainties during hydrogel injection. First, although there is a wide application of similar hydrogel spacer placement reported for esophagus, bladder, prostate, and cervix (42–44), we have limited experience in placing this hydrogel in unique C-loop anatomy at the interface between pancreas and duodenum. It’s hard for physicians to find an optimal injected point to place spacer only rely on 2D EUS images which might limit the benefit of hydrogel application. Second, the three-dimensional geometric relationship between the HOP and the duodenum can potentially change and deform since the beginning of the injection process. Similarly, 2D EUS images are not capable to represent all these deformations and changes in three-dimensional view for guiding the injection process. Our lab’s previous research (41) proposed a FEMOSSA simulation model to predict and simulate the realistic prostate-rectum spacer placement procedure. This method made it possible to design a pre-treatment injected plan based on CT scans for increasing the robustness and success rate of hydrogel placement, thereby potentially improving the clinical outcome of prostate cancer radiation therapy. Therefore, by combining the proposed method in this study with FEMOSSA, one can guide the EUS probe placing in the designed injected location and execute a pre-treatment injected plan.

The reason why the dictionary-based method of tracking probe position could be feasible and translated to our EUS guidance hydrogel injection is that probe motion pattern exists when the hydrogel is placed from the perspective of the duodenal lumen into the peripancreatic region. This kind of probe motion pattern also exists in clinical US image-guided procedures of prostate biopsy, cervical brachytherapy, and liver focal ablation (31). Plus, researchers demonstrated that Haar wavelet coefficients are sufficient and efficient to represent image features in 2D image slices to 3D volume image registration. In this case, abundant predicted EUS probe position of injection procedure is critical to generate efficient simulated EUS images and a large corresponding Haar features dictionary.

The results of the phantom and patient’s experiment show the feasibility of our method and the accuracy of finding the best matched simulated EUS image. Previous research (45, 46) showed that registration error within 3 mm is comparable with electromagnetic and vision-based tracking systems for spine needle injections in the lumbar region. However, our phantom results demonstrated that the error between the best matched simulated EUS image and target EUS image is within 1 mm. Additionally, the results demonstrated that Haar features are sensitive to detect targets even with a noisy background. By incorporating the integral image method, the computation procedure is not time-consuming. Our Haar feature method makes it possible to implement the proposed ideal injection workflow for reducing the risks caused by uncertainties in the injection process.

Our method does have a good performance for searching best matched simulated EUS images within the simulated EUS image set in the phantom experiment: every 2D simulated EUS image set includes 121 simulated images (rotation range: 30 degrees in 15 intervals, image plane interval: 5 mm). This is because the EUS training phantom only includes the simplest sphere targets. But we cannot find other EUS training phantom with more various targets to mimic the endoscopic injected process. In the patient MRI experiment, a searching error with our method occurred. This is probably because of the lower resolution of the simulated EUS image dictionary. Thus, if we aim to apply this method to EUS images of the human anatomy, it is better to refine the simulated EUS images in both fine rotated intervals and image plane intervals and improve EUS image quality. However, in this way, the simulation process will require more time.

There are several limitations of our study. The first limitation involves the fact that only one real-time EUS image is available to use as a target image for evaluating our results in the phantom experiment. Therefore, we do not have enough points to quantify the registration error. In more future work, some simulated EUS images could be viewed as target images to test our method. In addition, we could use them with different resolutions or in different directions to mimic the various circumstances in the actual clinical injection process. The advantage of the second solution is that we know the ground truth of the probe/image locations. Furthermore, we could collect various real EUS images with high resolution and image quality, extracting the Haar features, which are sensitive to edge detection, to train an auto-segmentation model, like face detection. Alternatively, we could use the results of an auto-segmentation of the pancreas, the pancreas duct, and vessels as targets to register CT scans with a real-EUS image.

Secondly, in phantom experiments, we only consider the endoscopic probe direction aligned with the scan channel. The endoscopic probe has broad flexibility in terms of rotation when injecting hydrogel within the duodenum. In that our sphere target has the same 2D projection in a different direction, the only difference is in its radius. However, if we implement this method in actual patient’s CT scans and EUS images, we have to consider more variations in probe direction. One previous paper (27) developed an imaging process method to generate potential/optimized planes for registration between CT and US images, which is a potential method we could combine with ours. In our patient experiment with CT scans, there is a large variation of interpolated CT slices with slightly “rotating” the probe. Thus, only 10 potential injection points are not sufficient to generate a simulated EUS dictionary. A similar limitation occurred in the patient’s MRI experiment. Plus, generating a large, simulated EUS dictionary including sufficient predicted probe position is owing to the EUS image simulation on Field II which is a very time-consuming process.

At last, our EUS training phantom CT has a low contrast resolution, and we do not have ground truth with our probe position. In future work, we could attach an infrared marker to the probe to track its location with an infrared camera. In this case, we could use this location data as ground truth to evaluate our results. There is a similar limitation to a patient experiment. Plus, the breathing motion effect was not considered when we did the simulation process based on patients’ CT and MRI. Since the EUS images were acquired in real time during clinical procedure, the motion breathing will probably cause no matched simulated EUS image in the dictionary even though the probe may be placed in the same position. In addition, many factors can impact the image quality and simulation process. For example, the grey level of region of interest in CT scan and EUS image are not uniform, such as stent, veins, and arteries. Some organs, like the layer of the mesentery, cannot be observed in CT scans, whereas these organs can be easily distinguished in the EUS images. Therefore, for generating a more accurate simulated EUS dictionary, additional image pre-processing steps that incorporate known anatomy are required.

## Conclusion

This study demonstrated the feasibility of our method for tracking endoscopic probe location without external tracking hardware, thereby guiding the hydrogel injection between HOP and duodenum. Ongoing studies aim to accelerate the simulation process of generating dictionaries. Furthermore, more variable potential injection points and EUS direction must be considered and included in the simulation process.

## Data Availability Statement

The raw data supporting the conclusions of this article will be made available by the authors, without undue reservation.

## Ethics Statement

The studies involving human participants were reviewed and approved by Johns Hopkins Medicine Institutional Review Boards (JHM IRBs). The patients/participants provided their written informed consent to participate in this study.

## Author Contributions

The study was designed by ZF, HH, and KD. All authors participated in collecting data. ZF and KD prepared the manuscript and contributed to data analysis and interpretation. All authors contributed to the article and approved the submitted version.

## Funding

Research reported in this publication was supported by the National Institutes of Health (award numbers R37CA229417).

## Author Disclaimer

The content is solely the responsibility of the authors and does not necessarily represent the official views of the National Institutes of Health.

## Conflict of Interest

The authors declare that the research was conducted in the absence of any commercial or financial relationships that could be construed as a potential conflict of interest.

## Publisher’s Note

All claims expressed in this article are solely those of the authors and do not necessarily represent those of their affiliated organizations, or those of the publisher, the editors and the reviewers. Any product that may be evaluated in this article, or claim that may be made by its manufacturer, is not guaranteed or endorsed by the publisher.

## Abbreviations

BR/LAPC, Borderline resectable or locally advanced pancreatic cancer; IMRT, Intensity modulated radiation therapy; SBRT, Stereotactic body radiation therapy; HOP, Head of pancreas; OAR, Organs at risk; EUS, Endoscopic ultrasound; CT, Computed tomography; MRI, Magnetic resonance imaging; TRUS, Transrectal ultrasound; NCC, Normalized cross correlation; ICT, Interpolated computed tomography; S-MRI, Simulated-magnetic resonance imaging; O-MRI, Original-magnetic resonance imaging.
